# Polymorphism analysis and expression patterns of the *IGF1* gene in the Shitou goose

**DOI:** 10.5194/aab-64-315-2021

**Published:** 2021-07-22

**Authors:** Jun Tang, Mao Guo, Jing Fu, Hongjia Ouyang, Yunbo Tian, Xu Shen, Yunmao Huang

**Affiliations:** 1 College of Animal Science & Technology, Zhongkai University of Agriculture and Engineering, Guangzhou, Guangdong, 510225, P.R. China; 2 Guangdong Provincial Key Laboratory of Waterfowl Healthy Breeding, Guangzhou, Guangdong, 510225, P.R. China

## Abstract

Insulin-like growth factor 1 (*IGF1*) is one of the endocrine hormones that plays an important role in regulating growth and development of animals. In this study, polymorphism in the 5${}^{\prime }$UTR and 3${}^{\prime }$UTR coding region and of the *IGF1* gene was detected by DNA sequencing technology, and the abundance of *IGF1* mRNA in various tissues at three growth stages of the Shitou goose was determined by quantitative real-time polymerase chain reaction (qRT-PCR). Moreover, the differential expression of *IGF1* in various tissues between the Shitou goose and Wuzong goose was revealed. Two single nucleotide polymorphisms (SNPs) were found in the exon3 region of *IGF1* in the Shitou goose. *IGF1* mRNA was extensively expressed in various tissues of Shitou geese with high abundant expression in the liver, breast muscle and leg muscle at three growth stages. *IGF1* mRNA expression showed a trend of first increase and then decrease in the pituitary, liver, subcutaneous fat and abdominal fat tissues, but it decreased in the breast muscle and leg muscle of a Shitou goose with growing age. Expression of *IGF1* in the liver, leg muscle and pituitary tissues of the Shitou goose was significantly higher than that of the Wuzong goose. This provides a foundation for further study of regulatory mechanisms of *IGF1* in the growth and development of geese.

## 1 Introduction

China is the largest goose-producing and goose-consuming country in the world. In 2018, 530 million commercial geese were for sale, and the number of geese in China accounted for more than 93.3 % in the world (Hou, 2019). Goose meat products are low in fat and cholesterol but high in protein. The protein content of goose meat reaches as high as 22.3 %, but its fat content is low at only about 11 %, of which unsaturated fatty acid content accounts for more than 99 % of the total (Mary, 1989). It is recognized as a healthy food and tremendously loved by consumers. Goose breeding is a profitable business in the contemporary the poultry industry. Production of poultry meat is closely related to its growth traits. Growth traits are important economic traits in poultry industry, which are controlled by genes and the environment (Goto et al., 2019). Therefore, studies on genes related to poultry growth and development represent a hot topic of interest.

Insulin-like growth factor 1 (*IGF1*) is one of the endocrine hormones that plays an important role in regulating growth, development, metabolism and reproduction of animals (Herbert et al., 2000). *IGF1* was first discovered by Salmon and Daughaday in 1957 (Salmon and Daughaday, 1957). It is a single-chain polypeptide composed of 70 amino acids with a relative molecular weight of 7649D (Bortvedt and Lund, 2012). Studies have found that *IGF1* can stimulate and promote the differentiation of muscle cells to participate in the genesis of skeletal muscle (Florini et al., 1991, 1996). Growth and development of animal muscle are closely related to temporal and spatial expression of IGFs (Tilley et al., 2007). Bhattacharya et al. (2015) found that *IGF1* expression in muscle was highest on day 1 and day 28, whereas the lowest expression was on day 42 in chickens. Hu et al. (2015) studied the expression of the *IGF1* gene in the pectoralis major muscle and lateral gastrocnemius muscle of a Gaoyou duck and a Jinding duck with different growth rates at the embryonic stage and 7 d after hatching; they found that the expression level of *IGF1* in the pectoralis major muscle of a Gaoyou duck was significantly higher than that of a Jinding duck at 13 and 21 embryonic days and 7 d of age. *IGF1* mRNA expression in chicken liver, which is the main organ for *IGF1* mRNA expression (Peng et al., 1996), is positively correlated with body weight increase (Beccavin et al., 2001). Studies on the molecular mechanism of *IGF1* are mainly focused on other animals, but there are few on geese.

The Shitou goose is the largest goose species originating from *Anser cygnoides* in the world, which is from Raoping County, Guangdong Province, China. The average weight of a male Shitou goose can be up to 10.39 kg (Lin et al., 2003), with excellent growth performance. The Wuzong goose is the smallest goose species originating from *Anser cygnoides* in the world, which is from Qingyuan City, Guangdong Province, China. The weight of a male Wuzong goose is only 3–3.5 kg (Gong, 2008), which is less than one-third of the Shitou goose, and its growth performance is very different. In addition, Tu et al. (2006) found that Shitou geese and Wuzong geese were clustered into one group through an unweighted pair-group method with arithmetic means (UPGMA) cluster analysis. It is suggested that the genetic background of Shitou geese and Wuzong geese is similar. It is an ideal model for studying the growth and development of Chinese local geese. In this study, mRNA expression patterns of *IGF1* in the hypothalamus, pituitary, liver, breast muscle, leg muscle, subcutaneous fat and abdominal fat tissues of 0, 5 and 15-week-old Shitou geese were detected by qRT-PCR. Meanwhile, differences of *IGF1* mRNA expression in these seven tissues were compared between a Shitou goose and Wuzong goose at 5 weeks of age, and polymorphism of *IGF1* was screened and analyzed.

## 2 Materials and methods 

### 2.1 Ethics statement

Housing, management and care of the birds conformed to the guidelines of the Animal Care and Use Committee at Zhongkai University of Agriculture and Engineering (approval number 2020-012) and also in accordance with the Animal Protection Law of the People's Republic of China.

### 2.2 Experimental animals and sample collection

The test birds used in this experiment were large-sized Shitou geese and small-sized Wuzong geese from Qingyuan Jinyufeng Goose Industry Co., Ltd (Guangdong, China). All the birds used in this experiment were raised in the same batch; they were free to eat and drink and were supplemented with green grass as much as possible during feeding. Three male Shitou geese were selected and slaughtered at 0, 5 and 15 weeks of age, and three male Wuzong geese were selected and slaughtered at 5 weeks of age. After slaughtering, hypothalamus, pituitary, liver, breast muscle, leg muscle, subcutaneous fat and abdominal fat tissues were collected and quickly transferred to liquid nitrogen for RNA extraction and qRT-PCR. A total of 57 Shitou geese blood samples were collected and stored at -80${}^{\circ }$ for DNA extraction.

### 2.3 DNA and RNA isolation and first-strand cDNA synthesis

DNA was isolated from goose blood using a Blood Genomic DNA Extraction Kit (Solarbio, China) according to the manufacturer's protocol and diluted to 100 ng µL${}^{-1}$. Total RNA was extracted from each tissue using TRIzol reagent (Invitrogen, Carlsbad, CA, USA) according to the manufacturer's protocol. Purified total RNA was dissolved in RNAase-free water and quantified by Nanodrop 2000 (Thermo, USA) at 260 nm. The RNA (3 µg) was synthesized to cDNA using a PrimeScript™ RT reagent kit with a gDNA Eraser reverse transcription kit (TaKaRa, Japan) according to the manufacturer's protocol and then stored at -20 ${}^{\circ }$C for qRT-PCR analysis.

### 2.4 Design of primers, PCR and qRT-PCR

The *IGF1* gene consists of 5${}^{\prime }$UTR, four exons and 3${}^{\prime }$UTR. The primers were designed from the available goose *IGF1* sequence (GenBank accession no. NW_013185696.1) using Primer 5.0 software (Premier Biosoft International, Palo Alto, CA, USA). Moreover, primers for reference genes were also designed (Table 1).

**Table 1:** Primer information of *IGF1* and a reference gene in geese.

Primer	Amplified	Primer sequence (5${}^{\prime }$–3${}^{\prime }$)	Product	Annealing	Usage
name	region		length	temperature	
	or gene		(bp)		(${}^{\circ }$C)
P1	5${}^{\prime }$ UTR + exon1	F: CTCTAAATCCCTCTTCT	949	52	SNP detection
		R: AGCAGCCCTCCCTACAA			
P2	exon2	F: AGATCCCTTTGCCTGTC	805	54	SNP detection
		R: TTGGGTCTAGTGTATCCTTT			
P3	exon3	F: AAAATACTTGCCTACTACAC	865	55	SNP detection
		R: TACCCAGAAACAGGATAA			
P4	exon4	F: AATAGTTTCGGTATGGTAG	909	52	SNP detection
		R: ACTGCTGCTGTTACTGG			
P5	3${}^{\prime }$ UTR	F: CAGTTTGACATTCCCAGTA	1073	54	SNP detection
		R: TCCATCCACCTTATTGC			
P6	IGF1	F: CGGAAGACTTAAAAAAAACTAT	169	56	RT-PCR
		R: TGGGCTGGTTAAAACGTTCTGT			
β-actin	β-actin	F: CCATCTATGAGGGCTACGCT	232	56	RT-PCR
		R: GCTTCTCCTTGATGTCACGG			

The PCR reaction was set up with 30 µg of DNA template, 10 ng of each primer, 1.5 mM of MgCl${}_{2}$, 100 µM of each dNTP, 1× assay buffer and 0.25 U of Taq DNA polymerase (MBI Fermentas, Amherst NY, USA). PCR was performed with initial denaturation at 94 ${}^{\circ }$C for 5 min, 33 cycles of denaturation at 94 ${}^{\circ }$C for 30 s, annealing at a specific temperature (Table 1) and extension at 72 ${}^{\circ }$C for 30 s with a final extension at 72 ${}^{\circ }$C for 5 min.

The qRT-PCR analysis was performed in triplicate using an iTaqTM Universal SYBR® Green Supermix Kit (Bio-Rad, Hercules, CA, USA) on an ABI QuantStudio 5 Real-Time PCR System (ABI, CA, USA). The relative expression levels were determined using the 2${}^{-\Delta \Delta \mathrm{Ct}}$ method (Livak and Schmittgen, 2001) with β-actin as a control. The reaction conditions were as follows: 95 ${}^{\circ }$C for 10 min, 40 cycles of 95 ${}^{\circ }$C for 15 s and 56 ${}^{\circ }$C for 60 s.

### 2.5 Polymorphism screening and genotyping

A total of 20 samples of Shitou geese DNA diluted to the same concentration were randomly selected and mixed to construct a DNA pool. PCR products from the DNA pool were sequenced to screen for gene polymorphism. Genotype mutations of each sample were analyzed by sequencing performed by Shanghai Shenggong Biological Co., Ltd. A total of 57 Shitou geese were used for polymorphism analysis.

### 2.6 Data analysis

Lasergene software was used to analyze the alleles. Genotype and allele frequencies of the Shitou goose were calculated and Hardy–Weinberg equilibrium (HWE) was calculated by the SHEsis program (Shi and He, 2005). In addition, population indexes such as effective allele numbers (Ne), polymorphic information content (PIC) and heterozygosity (He) were calculated following Nei's methods (Nei and Roychoudhury, 1974) performed by PopGene (version 1.3.1, AB, Canada). Significant differences between two samples and multi-groups were analyzed by one-way analysis of variance (ANOVA) and least square means for multiple comparisons using the SPSS 22.0 software.

## 3 Results

### 3.1 Sequence variation and polymorphisms analysis

**Figure 1:**
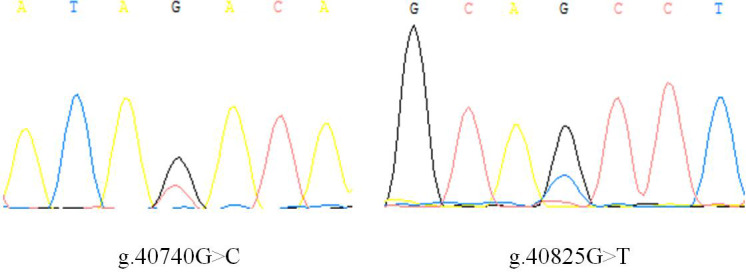
Sequencing results of PCR products from the DNA pool of the Shitou goose.

**Figure 2:**
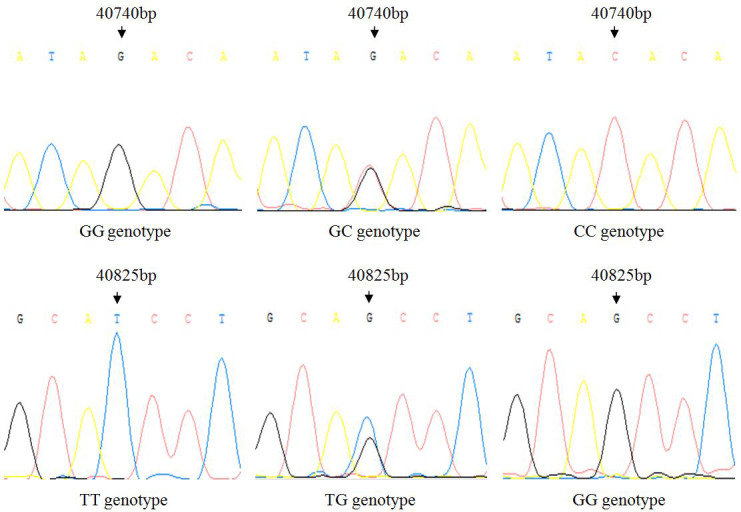
Sequencing results of three genotypes of two SNP loci.

There is no variation except two SNPs in the exon3 region of *IGF1*, in which there are nucleotide substitutions at positions 40 740 and 40 825 (Fig. 1), named as g.40740G>C and g.40825G>T. Three genotypes of two SNPs detected by sequencing are shown in Fig. 2.

Results of genetic polymorphism analysis of two SNPs are shown in Table 2. The major allele of g.40740G>C locus was G, with a frequency of 74.56 %. The major genotype was GG, with a frequency of 54.39 %. The g.40740G>C locus was moderately polymorphic (0.25< PIC <0.5) in Hardy–Weinberg equilibrium ($\chi^{2}>0.05$). The major allele of g.40825G>T locus was G, with a frequency of 70.18 %. The major genotype was TG, with a frequency of 56.14 %. The g.40825G>T locus was moderately polymorphic (0.25< PIC <0.5) in Hardy–Weinberg disequilibrium ($\chi^{2}<0.05$).

**Table 2:** Genetic diversity of two SNP loci in exon3 of *IGF1* in the Shitou goose.

Number	Genotypic frequency (%)			Allelic frequency (%)		Genetic polymorphism			Hardy–Weinberg
									equilibrium ($\chi^{2}$)
g.40740G>C									
	GG	GC	CC	G	C	Ne	He	PIC	
57	54.39	40.35	5.26	74.56	25.44	1.065	0.379	0.307	0.891
g.40825G>T									
	TT	TG	GG	T	G	Ne	He	PIC	
57	1.75	56.14	42.11	29.82	70.18	1.492	0.419	0.331	0.036

### 3.2 Tissue expression of *IGF1* in a 0-week-old Shitou goose

**Figure 3:**
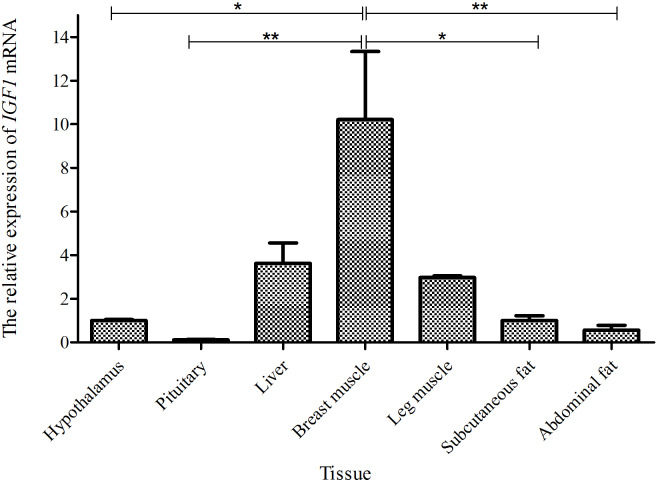
The mRNA expression of *IGF1* in different tissues of a 0-week-old Shitou goose. The ${}^{*}$ symbol represents a significant difference (P<0.05). The ${}^{**}$ symbol represents a very significant difference (P<0.01). The same applies to Figs. 4 to 7. Values are shown as the mean ± SD (n=3); the same applies to Figs. 4 to 7.

Taking the expression level of *IGF1* in the hypothalamus of a 0-week-old Shitou goose as a reference, the relative expression level of *IGF1* in seven different tissues of a 0-week-old Shitou goose is shown in Fig. 3. *IGF1* mRNA was ubiquitously expressed in all examined tissues (hypothalamus, pituitary, liver, breast muscle, leg muscle, subcutaneous fat and abdominal fat) with different abundances. Expression of *IGF1* in breast muscle was highest, which was significantly higher than that in the hypothalamus and subcutaneous fat (P<0.05), as well as the pituitary gland and abdominal fat (P<0.01). The second-highest expression of *IGF1* was in liver and leg muscle, while the lowest was in the pituitary gland. It is suggested that *IGF1* is possibly related to muscle growth and development of the Shitou goose at the time of hatching.

### 3.3 Tissue expression of *IGF1* in a 5-week-old Shitou goose

**Figure 4:**
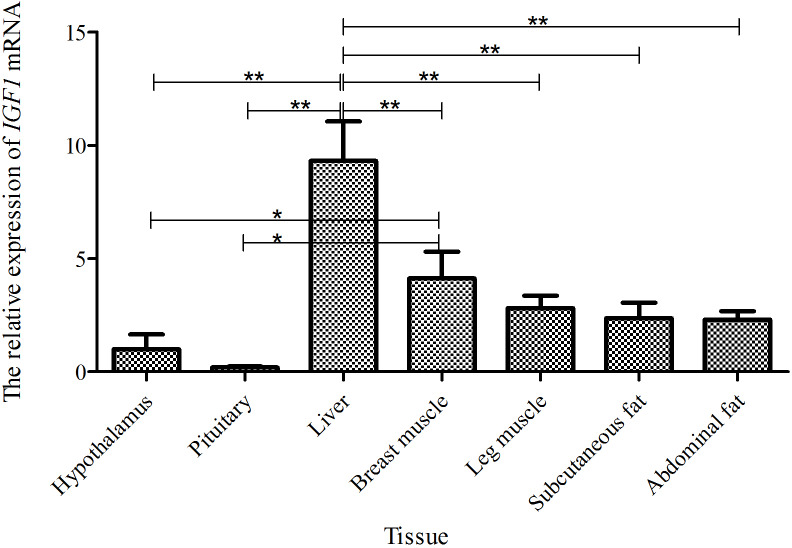
The mRNA expression of the *IGF1* gene in different tissues of a 5-week-old Shitou goose.

Taking the expression level of *IGF1* in the hypothalamus of a 5-week-old Shitou goose as a reference, the relative expression level of *IGF1* in seven different tissues of a 5-week-old Shitou goose is shown in Fig. 4. The expression level of *IGF1* in the liver was highest, which was significantly higher than that in other tissues (P<0.01). The expression level of the *IGF1* gene in breast muscle was significantly higher than that in the hypothalamus and pituitary tissue. Expression in leg muscle, subcutaneous fat and abdominal fat was also higher. It is suggested that *IGF1* probably has an influence mainly on functions in the liver and the growth and development of muscle and adipose tissue at 5 weeks of age.

### 3.4 Tissue expression of *IGF1* in a 15-week-old Shitou goose

**Figure 5:**
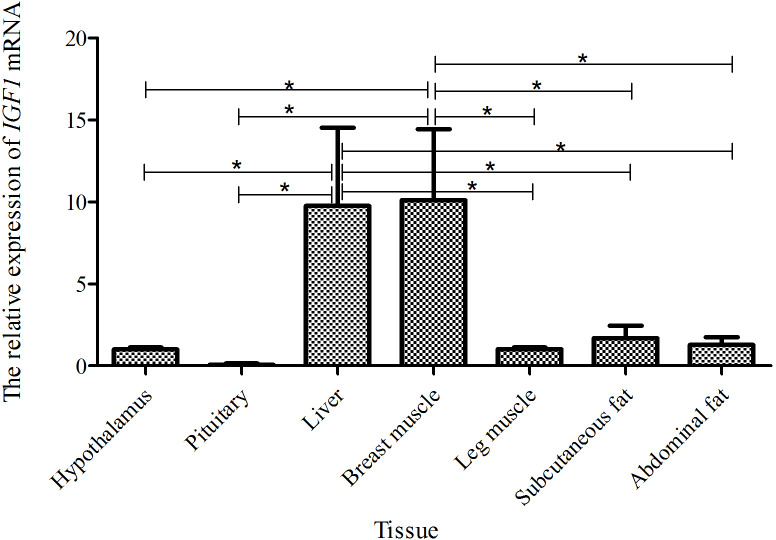
The mRNA expression of the *IGF1* gene in different tissues of a 15-week-old Shitou goose.

Taking the expression level of *IGF1* in the hypothalamus of a 15-week-old Shitou goose as a reference, the relative expression level of *IGF1* in seven different tissues of a 15-week-old Shitou goose is shown in Fig. 5. The expression level of *IGF1* in breast muscle was highest, which was significantly higher than that in other tissues except the liver. Expression of *IGF1* in subcutaneous fat, abdominal fat, hypothalamus, leg muscle and pituitary tissue was low, and the difference was not significant between them.

### 3.5 Expression patterns of the *IGF1* gene in the same tissue at different weeks of Shitou geese 

**Figure 6:**
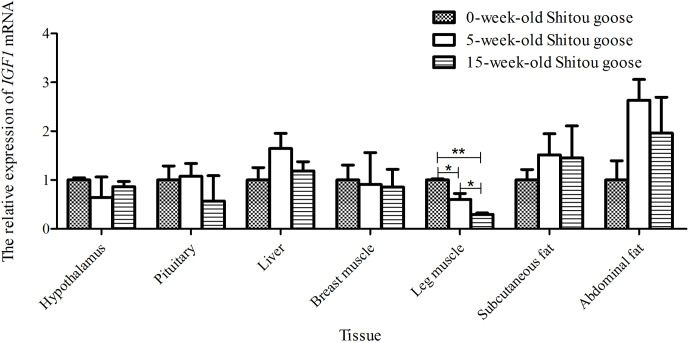
The mRNA expression of the *IGF1* gene in three periods for a Shitou goose.

Taking the expression level of *IGF1* in a 0-week-old Shitou goose in different tissues as a reference, the relative expression level of *IGF1* in the same tissue at different growth stages is shown in Fig. 6. Expression of *IGF1* in pituitary, liver, subcutaneous fat and abdominal fat tissues of a Shitou goose showed a trend of first increase and then decrease with the increase in age. However, the expression of *IGF1* in the hypothalamus showed a trend of first decrease and then increase with the increase in age. Expression of *IGF1* in breast muscle and leg muscle decreased with the increase in age. It can be seen that the expression of *IGF1* in different stages of the same tissue is quite different.

**Figure 7:**
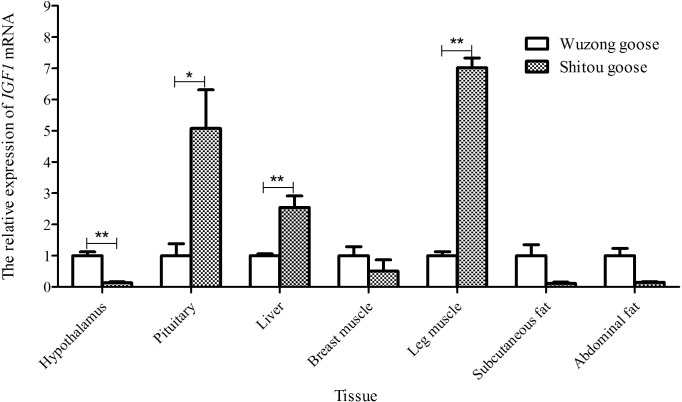
Differential expression of the *IGF1* gene in different tissues between a 5-week-old Shitou goose and Wuzong goose.

### 3.6 Differential expression analysis of *IGF1* in different tissues of a 5-week-old Shitou goose and Wuzong geese

Taking the expression level of *IGF1* in a 5-week-old Wuzong goose as a reference, the differential expression of *IGF1* in different tissues of a 5-week-old Shitou goose and Wuzong goose is shown in Fig. 7. The expression level of *IGF1* in the liver and leg muscle tissues of a 5-week-old Shitou goose was significantly higher than that of a Wuzong Goose (P<0.01), and the expression level of *IGF1* in the pituitary tissue of a 5-week-old Shitou goose was significantly higher than that of a Wuzong goose (P<0.05). Expression of the *IGF1* gene in the hypothalamus of a 5-week-old Shitou goose was significantly lower than that of a Wuzong goose (P<0.05).

## 4 Discussion

*IGF1* is an important growth regulator, which plays a vital role in animal growth and development through autocrine and paracrine functioning (Bondy and Cheng, 2004). Therefore, the study of *IGF1* gene polymorphism and expression patterns is helpful to understand the regulatory mechanism of *IGF1* in animal growth and development.

The regulation of *IGF1* in animal growth and development has established a relationship between polymorphism and growth performance. Bian et al. (2008) identified three SNPs (c.-366A>C, c.528G>A and c.${}^{*}$1024C>T) that constructed haplotypes in the chicken *IGF1* gene. Haplotypes based on three polymorphisms were associated with body weight traits. Sato et al. (2011) revealed that a single nucleotide polymorphism (g.570C>A) in *IGF1* was significantly associated with breast muscle yields in the F2 population for which it was an intercross between a high-breast-muscle-yield male line and a low-breast-muscle-yield female line in chickens. In this study, two SNPs were found in the exon3 region of the *IGF1* gene in Shitou geese. The PIC of two SNPs was moderate, and the G-40740C locus was in a Hardy–Weinberg equilibrium state. The proportion of homozygous CC (g.40740G>C) and TT (g.40825G>T) is very low, but the proportion of heterozygosity is about 50 %. The potential reason may be the Shitou goose undergoing artificial selection during evolution. It provided a foundation for revealing the relationship between goose growth performance and *IGF1* gene polymorphism, and it also provided some reference for goose breeding.

In this study, it was found that *IGF1* mRNA was expressed in the hypothalamus, pituitary gland, liver, breast muscle, leg muscle, subcutaneous fat and abdominal fat at three growth stages of 0, 5 and 15 weeks of age. Expression of *IGF1* in the liver was high in all three growth stages. Similar results have also been reported in Jinghai yellow chickens (Abdalhag et al., 2016) and pigs (Peng et al., 1996), which indicates that the liver is the major endocrine source of IGFs (Zahran and Aboul-Soud, 2008; Georgiev, 2010). *IGF1* is also a kind of high-performance muscle nutrition factor, which plays an important role in animal protein deposition and muscle cell proliferation. Its expression in muscle can significantly promote growth and development of muscle (Mitchell, 2002; Coleman, 1995). In the three growth stages of this study, expression of *IGF1* was always higher in the breast muscle and leg muscle except for the leg muscle at 15 weeks of age, indicating that the expression of *IGF1* may promote muscle growth and development in the Shitou goose.

With the increase in age, expression of *IGF1* showed different patterns in different tissues. Expression of *IGF1* in the pituitary, liver, subcutaneous fat and abdominal fat tissues of a Shitou goose showed a trend of first increase and then decrease, with the highest at 5 weeks of age. However, 5 weeks of age is the high-speed growth and development period of a Shitou goose (Lu et al., 2002). The results showed that the expression of *IGF1* was higher in most tissues during the rapid growth and development period, indicating that *IGF1* probably plays a role in both growth and development of geese. This is similar to other studies in that a highly variable relationship exists between circulating *IGF1* and post-hatch growth in chickens (Vasilatos-Younken and Scanes, 1991), and a positive correlation (r=0.62) exists between circulating concentrations of *IGF1* and body weight (Goddard et al., 1988). *IGF1*, the expression of which is increased during the differentiation of human preadipocytes, can also promote the proliferation and differentiation of preadipocytes (Kelly et al., 2010; Bouraoui et al., 2010; Susann et al., 2005), and both preadipocytes and adipocytes can secrete *IGF1* (Hemkendreis, 2000). Therefore, the high expression of *IGF1* in adipose tissue can promote the growth and development of adipose tissue. However, the expression of *IGF1* in breast muscle and leg muscle decreased gradually with aging, which was similar to a study conducted by Peng et al. (1996), who reported a decrease in the expression of the *IGF1* gene in pig skeletal muscle (Peng et al., 1996). At the same time, it may be related to the earlier development of breast and leg muscles. Yun et al. (2005) found that the breast muscle gains weight most rapidly in 1–3 weeks, which was more than 5 times the birth weight. Therefore, *IGF1* expressed abundantly in early stages promotes the rapid growth of muscle.

There are great differences in growth traits between Shitou geese and Wuzong geese, which is an ideal model for studying bird growth and development. In this study, the expression of *IGF1* in liver and leg muscle tissues of a Shitou goose was significantly higher than that of a Wuzong goose, and the expression level of the *IGF1* gene in pituitary tissue of a Shitou goose was significantly higher than that of a Wuzong goose. Liver and muscle are the main organs for *IGF1* secretion and function; *IGF1* mRNA expression in chicken liver is positively correlated with body weight increase (Beccavin et al., 2001), and the expression of *IGF1* in muscle can noticeably promote the growth and development of muscle (Mitchell et al., 2002; Coleman et al., 1995). Hence, it is indicated that *IGF1* may play an important role in regulating the rapid growth of Shitou geese.

## 5 Conclusions

In this study, two SNPs were found in the exon3 region of the *IGF1* gene in Shitou geese. *IGF1* mRNA expression was higher in most tissues during the rapid growth and development period and was extensively expressed in various tissues of a Shitou goose with different abundances at all three growth stages. Expression of *IGF1* in the liver, leg muscle and pituitary tissues of a Shitou goose was very significantly or significantly higher than that of a Wuzong goose, indicating that the *IGF1* gene may play an important role in regulating the growth and development of geese.

## Data Availability

The original data of the paper are available from the corresponding author upon request.
